# Growth and Nutritional Quality of Lemnaceae Viewed Comparatively in an Ecological and Evolutionary Context

**DOI:** 10.3390/plants11020145

**Published:** 2022-01-06

**Authors:** Barbara Demmig-Adams, Marina López-Pozo, Stephanie K. Polutchko, Paul Fourounjian, Jared J. Stewart, Madeleine C. Zenir, William W. Adams

**Affiliations:** 1 Department of Ecology and Evolutionary Biology, University of Colorado, Boulder, CO 80309, USA; Stephanie.Polutchko@Colorado.EDU (S.K.P.); paul@internationallemnaassociation.org (P.F.); Jared.Stewart@Colorado.EDU (J.J.S.); Madeleine.Zenir@colorado.edu (M.C.Z.); William.Adams@colorado.edu (W.W.A.III); 2 Department of Plant Biology and Ecology, University of the Basque Country (UPV/EHU), 48049 Bilbao, Spain; marinalopezpozo@hotmail.com; 3 International Lemna Association, Denville, NJ 07832, USA

**Keywords:** chlorophyll fluorescence, electron transport chain, inflammation, lutein, photosystem, photosynthetic capacity, relative growth rate

## Abstract

This review focuses on recently characterized traits of the aquatic floating plant *Lemna* with an emphasis on its capacity to combine rapid growth with the accumulation of high levels of the essential human micronutrient zeaxanthin due to an unusual pigment composition not seen in other fast-growing plants. In addition, *Lemna*’s response to elevated CO_2_ was evaluated in the context of the source–sink balance between plant sugar production and consumption. These and other traits of Lemnaceae are compared with those of other floating aquatic plants as well as terrestrial plants adapted to different environments. It was concluded that the unique features of aquatic plants reflect adaptations to the freshwater environment, including rapid growth, high productivity, and exceptionally strong accumulation of high-quality vegetative storage protein and human antioxidant micronutrients. It was further concluded that the insensitivity of growth rate to environmental conditions and plant source–sink imbalance may allow duckweeds to take advantage of elevated atmospheric CO_2_ levels via particularly strong stimulation of biomass production and only minor declines in the growth of new tissue. It is proposed that declines in nutritional quality under elevated CO_2_ (due to regulatory adjustments in photosynthetic metabolism) may be mitigated by plant–microbe interaction, for which duckweeds have a high propensity.

## 1. Introduction

The smallest known flowering plants are found in the Lemnaceae family and are recognized (see recent comprehensive review by Acosta et al. [1]) for their attractive combination of extremely high growth rates [2,3] with high nutritional quality, including a high protein content, with all essential amino acids for humans, as well as a high content of essential human micronutrients [4]. The Lemnaceae, commonly known as duckweeds, water lentils, or water lenses, are comprised of five genera, including *Lemna* and *Wolffia* [1]. The high protein content of Lemnaceae is associated with their propensity for the efficient uptake and accumulation of nitrogen and other mineral nutrients, which makes them good at wastewater recycling [5,6,7,8,9] and contributing to a high nitrogen-use efficiency in agricultural contexts [10].

Additional traits of interest include duckweed’s ability to accumulate high levels of starch as well as their relatively low susceptibility to the undesirable effects of elevated atmospheric carbon dioxide levels (for details, see sections dedicated to these topics below). Moreover, duckweed’s rapid growth and diminutive size allow for a high volumetric yield in tight quarters such as greenhouses, urban rooftop growth facilities, and spacecrafts, where duckweed’s insensitivity to microgravity is another boon [1,11]. Our review places the above traits, as well as additional traits recently described by our group, into the context of the ecology and evolution of Lemnaceae in comparison with terrestrial plants as well as other floating aquatic plants.

The traits of *Lemna* recently characterized by us, which are the focus of this review, include:

- The pronounced tolerance of a wide range of growth light intensities and a remarkable capacity to accumulate high levels of antioxidant micronutrients, such as the essential carotenoid zeaxanthin, due to an unusual pigment composition not seen in fast-growing land plants.
- The response to elevated CO_2_ as evaluated in the context of plant metabolic regulation of the source–sink balance (balance between the plant’s sugar production and consumption), carbon-to-nitrogen ratio, and redox homeostasis (balance of oxidants and antioxidants).

## 2. Exceptions to Common Trade-Offs: Araceae and Lemnaceae

### 2.1. Trade-Off between the Ability to Grow in Deep Shade and Full Sun

Many land plants exhibit a trade-off between the ability to tolerate deeply shaded growth environments on the one hand and extremely high light levels on the other. Specifically, fast-growing species are typically sun-loving and unable to grow in deep shade. Exceptions are found in the family Araceae, which belongs to the same order (Alismatales, water plantains) as the Lemnaceae. The Alismatales include many floating or submersed aquatic and wetland species found in marshy and marine habitats [12]. Araceae is the most species-rich family of the Alismatales and is remarkable for the highly diverse habitats in which its species thrive, ranging from open freshwater to deserts, and its diverse life forms, including hemi-epiphytes, epiphytes, terrestrial species, and aquatic plants [13]. It has been noted that some Araceae exhibit a high level of flexibility in the organization of the photosynthetic membrane [14] (see also below).

Plants can suffer damage from intense light unless they process absorbed light either in photosynthesis (which converts excitation energy to chemical energy) or via safe alternative processes (that, e.g., convert excitation energy to harmless thermal energy; see [15,16]). Many species in the Araceae can grow equally well in deep shade and in full sun (see [16,17]). For example, the hemi-epiphyte *Monstera deliciosa* (Araceae) germinates in deep shade on the rainforest floor, climbs the nearest tree, and eventually completes its life cycle in the sun-flooded forest canopy after shedding its connection to the soil. In full sun, *M. deliciosa* exhibits low rates of photochemical energy utilization but record rates of the alternative, non-photochemical dissipation of absorbed light as thermal energy [17]. This latter process is catalyzed by antioxidants with critical roles in fighting radiation damage in both plants and humans (for details, see sections below).

### 2.2. Common Trade-Off between Fast Growth and Antioxidant Accumulation

**Figure 1:**
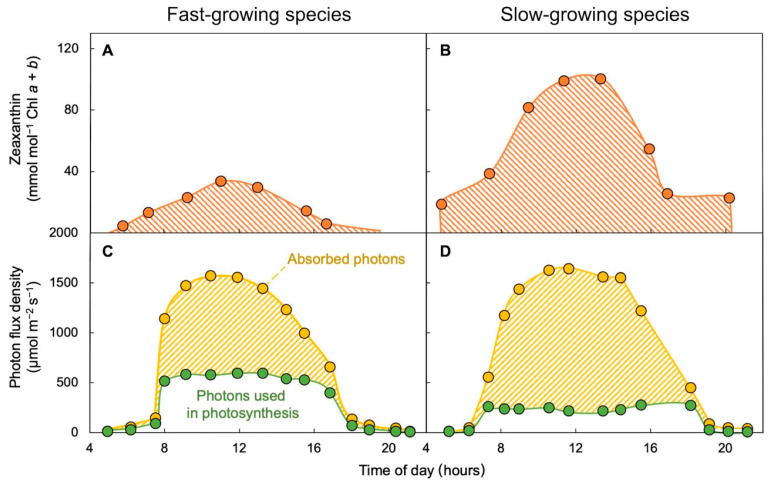
Diurnal time course of changes in (**A**,**B**) zeaxanthin level and (**C**,**D**) photons absorbed and utilized through photosynthetic electron transport in (**A**,**C**) the fast-growing annual crop *Helianthus annuus* (sunflower) versus (**B**,**D**) the slow-growing perennial *Vinca major*. Data from [18]; re-drawn from [16].

Terrestrial plants typically show a trade-off between growth rate and the accumulation of radiation-fighting antioxidants, especially the carotenoid zeaxanthin [16]. Fast-growing terrestrial species tend to accumulate less zeaxanthin, whereas slow-growing species tend to accumulate more zeaxanthin (Figure 1; [16,18]). This difference in response is the expected result of the above-described link to the fraction of absorbed light processed in photosynthesis. Fast-growing species use a greater fraction of the light they absorb at peak irradiance to support growth and accumulate less of the antioxidant zeaxanthin, which harmlessly removes light not usable for growth, i.e., excess light [16,18]. Conversely, slow-growing species (e.g., *M. deliciosa*) use a lesser fraction of absorbed light for growth and accumulate more zeaxanthin (Figure 1; [16,18]). The Araceae are thus no exception to the general rule of a trade-off between fast growth and accumulation of high levels of zeaxanthin [17,19].

### 2.3. Zeaxanthin—Essential Human Micronutrient and Hard to Come by in the Diet

The essential micronutrient zeaxanthin is not easy to come by in the human diet for the very reason that leaves produce zeaxanthin to fight radiation damage only when necessary. The green parts of plants (such as leafy greens) only accumulate zeaxanthin when they need to actively dispose of excessive, potentially damaging light and quickly remove the dissipator zeaxanthin as soon as light levels drop. This dynamic process gives plants the advantage of allowing photosynthesis to return to highly efficient utilization of absorbed light (Figure 1; reviewed in [16]). Food sources of zeaxanthin, other than leafy greens, include egg yolks and yellow corn, which owe their color to the presence of zeaxanthin and the closely related xanthophyll lutein (see, e.g., [20]). However, neither eggs nor corn are particularly suitable for production in limited spaces. In particular, operation of a chicken farm or corn field is not feasible on a spacecraft and may also not be an attractive approach in an urban setting or a greenhouse.

**Figure 2:**
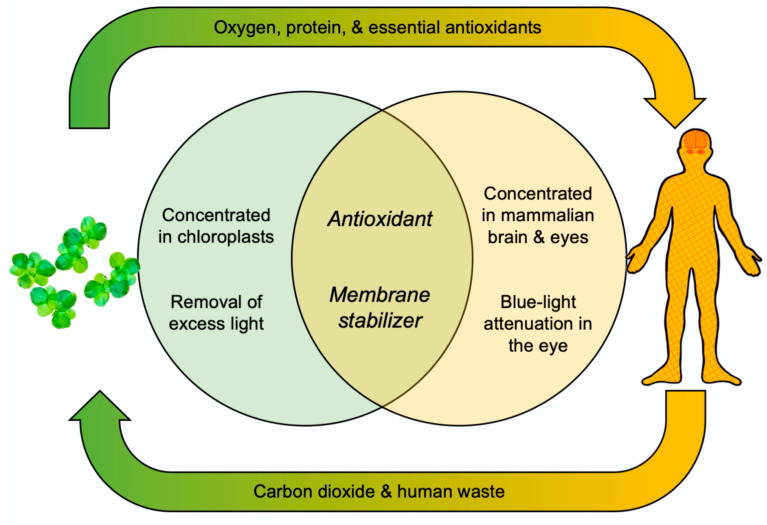
Schematic depiction of the functions of zeaxanthin in plants and humans with both overlapping and analogous aspects.

The essential dietary carotenoid zeaxanthin is not only needed to fight radiation damage and system-wide inflammation in humans but also to support mental acuity in healthy young adults (Figure 2; [21,22]). Zeaxanthin enhances basic membrane function in the brain, which supports mental acuity and detoxifies free radicals and other oxidants, which lessens radiation damage and the system-wide inflammation linked to less-than-optimal function, disorders, and diseases. Additional essential micronutrients act synergistically with zeaxanthin in fighting radiation damage and inflammation; zeaxanthin thus works best in a total package—provided by whole foods—with additional plant antioxidants that recycle zeaxanthin for a longer lifetime in inflammation fighting (Figure 2; [22]). Figure 2 places antioxidants with overlapping essential functions in plants and humans into the cycle of oxygen and carbon dioxide exchange, organic nutrients (e.g., protein and antioxidants), and inorganic nutrients (human waste) between photosynthetic and non-photosynthetic organisms. Thereby, Figure 2 highlights that duckweed is an excellent plant component for a regenerative life support system on, e.g., a spacecraft (see also [23,24]).

As reviewed in Demmig-Adams et al. [21], airline pilots (exposed to ionizing radiation) who reported eating more zeaxanthin exhibited less inflammation; similarly, healthy young subjects given zeaxanthin for six months showed less inflammation and enhanced cognitive performance in complex tasks, greater speed at completing those tasks, and better memory and attention. Sufficient dietary zeaxanthin would thus appear to be especially critical for long-duration human spaceflight. As further illustrated in the following section, duckweed provides a renewable food source that can be grown in limited space and can be exceptionally rich in zeaxanthin.

### 2.4. Lemna: An Unusual, Fast-Growing Hyperaccumulator of Zeaxanthin

Among land plants, it is the relatively slow-growing evergreens that typically exhibit the highest zeaxanthin concentrations [16,25]. An extreme example is evergreen conifers that arrest growth completely when they overwinter at high altitude where soil water freezes in the winter. While green needles can absorb a lot of light, they dissipate 100% of it as thermal energy via zeaxanthin as long as water lost from the needles during CO_2_ uptake for photosynthesis cannot be replaced [26,27]. As stated above, fast-growing terrestrial annuals instead typically utilize a significant amount of the light they absorb in photosynthesis and do not dissipate as much. When subjected to environmental stresses that can curb their growth rate, such as limiting nutrient levels in the soil [28,29], annuals typically strongly downregulate chlorophyll levels (and thus light-harvesting capacity) and only exhibit moderate increases in zeaxanthin levels employed in energy dissipation. When grown under extreme light conditions (with respect to high intensity and long photoperiod) that resulted in a comparably low chlorophyll content per area, *L. gibba* accumulated significantly more zeaxanthin [23,24] than spinach grown under limiting nutrient supply [29,30].

**Figure 3:**
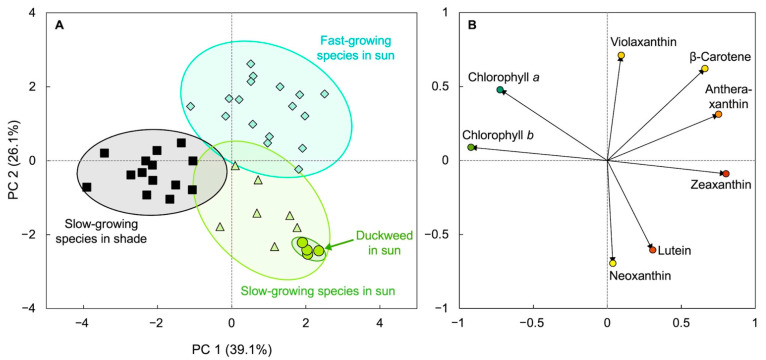
Principal component analysis showing (**A**) clusters of groups based on similarity (score plot) and (**B**) how each characteristic used influences a principal component, PC (loading plot). Characteristics used were the various leaf pigments of slow-growing terrestrial species either in shaded environments (black squares) or full sunlight (olive-green triangles), fast-growing (annual and biennial) species (turquoise diamonds) in full sunlight, and duckweed (*Lemna minor*) that was growing in full sunlight (olive-green circles) near Boulder, CO, USA (for details, see [24,25]). Data from [24,25].

Further characterization of *Lemna*’s exceptionally strong zeaxanthin accumulation led to the identification of a carotenoid composition that is unusual for fast-growing plants (Figure 3). A comparison of *Lemna* with several terrestrial species via principal component analysis showed that *Lemna* fell into the olive-green ellipse of slow-growing plants with high levels of zeaxanthin (and lutein) rather than the blue-green ellipse of other fast-growing plants (Figure 3; [24,25]). In other words, *Lemna* combines an exceptionally high growth rate with an unusual carotenoid composition and accumulation of as much zeaxanthin as seen in slow-growing land plants (see below).

**Figure 4:**
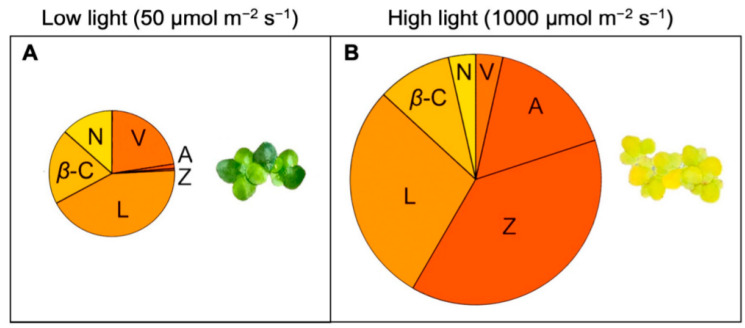
Carotenoid composition of *Lemna gibba* grown under continuous (**A**) low or (**B**) high light intensity. The size of the respective pies represents the molar ratio of the sum of all carotenoids to chlorophyll. Data from [24]; adapted from [16]. A, antheraxanthin, β-C, β-carotene, L, lutein, N, neoxanthin, V, violaxanthin, Z, zeaxanthin.

Once again, the traits of duckweed are thus a mix of those seen in slow-growing terrestrial evergreens (with greater maximal zeaxanthin levels) and fast-growing terrestrial annual plants (with lower maximal zeaxanthin levels). Future research is needed to confirm that the high growth rate and the unusually pronounced accumulation of zeaxanthin in *Lemna* (Figure 4) is associated with thin leaves that likely cause a relatively greater portion of the frond cross-section to be exposed to high light when plants are grown under a high growth light intensity. Terrestrial plants often feature multi-layered canopies, of which only the top leaves receive unfiltered light. Additionally, terrestrial plants typically also feature multi-layered leaves, where only the top cell layer receives unfiltered light. When grown in full sun, leaves of terrestrial plants thus exhibit pronounced high-light acclimation [31], with the highest levels of xanthophylls, and especially zeaxanthin, in their top-most layers [32]. In contrast, duckweed and other floating plants consist of relatively thinner leaves/fronds that presumably experience less attenuation of light from top to bottom. This scenario is supported by the side-by-side comparison of a terrestrial weed with duckweed [24] growing in a high-light exposed location, where the terrestrial weed exhibited a much higher chlorophyll level per unit leaf area typical for that seen in leaves with multiple chloroplast-rich layers of mesophyll cells.

One can think of duckweed fronds as structures that allow a thin layer of chloroplasts to be uniformly exposed to unfiltered light, which may require unusually high levels of excess-light dissipation and zeaxanthin accumulation under exposure to high light intensities. Hypothetically, duckweed could simply lower its chlorophyll content (and antenna size) enough to avoid absorbing much excess light. However, the maintenance of sufficient chlorophyll levels, accompanied by strong protection from zeaxanthin as needed, may be advantageous to duckweeds that can rapidly shift between being at the top versus in a deeper layer when growing in dense mats.

In addition to being bound to specific designated sites in chlorophyll-binding light-harvesting complexes, additional zeaxanthin can be dissolved in the phospholipid bilayer of plants’ photosynthetic membranes outside of chlorophyll-binding proteins (as first reported in [33] and recently reviewed in [34]). The report of a ratio greater than 0.5 (mol/mol) for zeaxanthin to chlorophyll *a* + *b* [24] suggests that a substantial fraction of the zeaxanthin accumulated in golden-hued duckweed fronds (Figure 4) growing under continuous high light levels may be dissolved in these membranes rather than being bound to protein complexes. At the same time, this high zeaxanthin content is not associated with sustained lowering of photosystem II efficiency [23,24] as is seen in many plants (see [35] for *Arabidopsis thaliana* and [16] for evergreens) under environmental stresses. Such a scenario—high zeaxanthin levels uncoupled from sustained lowering of photochemical efficiency—is especially desirable for optimal human nutrition without plant productivity losses. Other aquatic floating flowering plant species (beyond Lemnaceae) have also been reported to exhibit very high levels of xanthophylls [36], although xanthophylls were not separated into zeaxanthin versus other di-hydroxy xanthophylls (like lutein) during these analyses. This possibility that other aquatic plants share the above-described features of duckweed is consistent with a report by Rice [37] that noted aquatic taxa possessed unique photosynthetic features, including pigment composition. Future research should assess these features of aquatic species, which could be of interest for human nutrition, e.g., with respect to zeaxanthin levels. It is noteworthy that many aquatic floating plants are described as edible and/or medicinal plants (see, e.g., [38]).

### 2.5. Remote Sensing of Duckweed Zeaxanthin Content and Biomass Production

**Figure 5:**
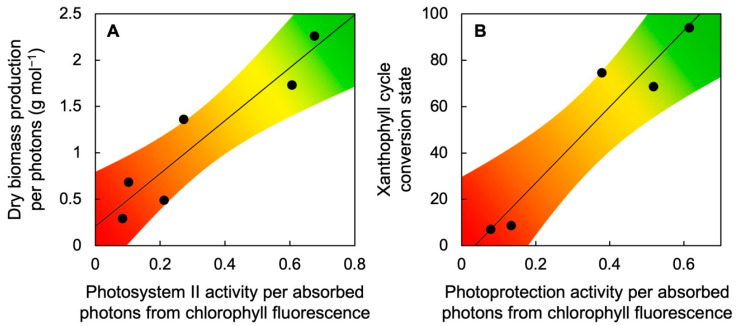
Relationship between (**A**) dry biomass production per photons and photosystem II activity per absorbed photons from chlorophyll fluorescence (as photosystem II efficiency from F_v_′/F_m_′ × q_P_; see [18,24]) or (**B**) xanthophyll cycle conversion state, (Z + A)/(V + A + Z), and the activity of non-photochemical dissipation of excess absorbed light from chlorophyll fluorescence (as photoprotective thermal dissipation from 0.8 − F_v_′/F_m_′; see [18,24]) under six different growth-light conditions over a range of light intensities from 50 to 1000 µmol photons m^−2^ s^−1^. Data from [23,24]. A, antheraxanthin, V, violaxanthin, Z, zeaxanthin.

Chloroplast-containing plant organs emit chlorophyll fluorescence, a signal that provides information about the fraction of absorbed light utilized in photosynthesis versus the fraction dissipated alternatively (non-photochemically) in the form of thermal energy. The fraction of absorbed light utilized in photosynthetic electron transport has been used, in conjunction with other parameters, to predict plant productivity (see, e.g., [39]). In terrestrial plants, this relationship is complex ([40,41]; see also the discussion in [24]). However, a simple, linear positive correlation exists in *Lemna* between this utilization of absorbed light as ascertained from chlorophyll fluorescence (as photosystem II activity per photons) and *Lemna* biomass production per photons (Figure 5A; [24]). In addition, the activity of non-photochemical, photoprotective energy dissipation assessed from chlorophyll fluorescence is positively and linearly correlated with xanthophyll cycle conversion to zeaxanthin (Figure 5B; [23,24]). Chlorophyll fluorescence measurements thus allow non-destructive, remote estimates of zeaxanthin level (see also [16]).

## 3. Comparative Evaluation of Adaptations in Aquatic and Terrestrial Plants

### 3.1. Rapid Growth in Duckweeds and Other Aquatic Plants

*Lemna* grows very fast [3] even under conditions of limited light supply [23,24]. We suggest that this unique feature may be associated with minimal self-shading within its relatively thin photosynthetic organs (fronds) and their single layer of fronds (no tiered structure) across the water surface, which should permit all chloroplasts to contribute significantly to sugar production. Other floating flowering plants also grow remarkably fast [42,43], which may be related to a high allocation of resources to photosynthetic tissue as discussed by Rice [37]. Terrestrial plants growing in soil must invest a sizeable fraction of their photosynthetically produced sugar into building (i) root structures to provide a stable anchor in the soil, for water (and nutrient) acquisition, and for the storage of carbohydrates and (ii) reinforced stem structures for support of the shoot system and transport of water, nutrients, and sugar between their photosynthetic organs and the roots. In contrast, floating plants have minimal non-photosynthetic tissue and can thus re-invest most of their photosynthate into more photosynthetic tissue. The main carbohydrate sink for duckweed is vegetative growth, its expanding daughter fronds that also contribute to photosynthetic productivity. Duckweed lacks typical stems or roots that would act as substantial sinks for photosynthate, and its roots can be green and contribute to photosynthesis. It has also been pointed out that Lemnaceae have undergone substantial genome reduction—including gene clusters that control growth [44] in response to the environment in terrestrial plants. Unabated growth of new tissue under a wide variety of environmental conditions is thus duckweed’s main sink activity.

### 3.2. Continuum of Plant Adaptations to Water Availability

Another perspective from which to view the fast growth of aquatic plants is their rather continuous access to water. Variable access to water is one of the major environmental variables for terrestrial plants. Many, but not all, terrestrial species curb stomatal opening, and growth, under limiting water as a defense strategy [45]. Such a response would appear less necessary for species that float on water, and duckweeds indeed impose much less control on either stomata or growth rate [44]. A review by Dolferus [46] entitled “To grow or not to grow: A stressful decision for plants” concludes that highly responsive modulation of growth rate—with either slower or faster growth—is one of the major ways for terrestrial plants to respond to changes in water availability. Table 1 compares the response of aquatic floating plants with the continuum of responses of different groups of terrestrial plants.

The high growth rate seen in many aquatic plants [42,43] is reminiscent of the high growth rates of terrestrial plants that maintain constant access to water throughout their life cycle (Table 1). Desert ephemerals are an example of terrestrial plants with an extremely high growth rate that rapidly complete their accelerated life cycle after infrequent major rainfall events in arid environments [47]. Other examples for terrestrial plants with high growth rates are annuals and biennials that also complete their life cycle in a relatively short period of time and grow where they can maintain access to water. These species may employ evasive approaches to keep internal water content high, e.g., by performing osmotic adjustment to maintain metabolic activity [48] and increasing their investment in water-mining root structures [49] (Table 1). These terrestrial plants do not put the brakes on growth until water becomes scarce, and then switch to seed production—reminiscent of the production of vegetative storage forms (turions) in Lemnaceae. Yet, other terrestrial plants, such as palm trees or mesquite, have deep roots that access the ground-water table [50,51,52,53,54], which supports steady growth despite low rainfall or moisture level in the air. Pronounced osmotic adjustment [55] and expansion of root volume [56] is also employed by terrestrial plants that grow very slowly in areas with extremely low water availability (Table 1).

Those terrestrial plants that tolerate—rather than evade—internal water deficits exhibit trade-offs between growth rate and stress tolerance/antioxidant content. In extreme natural environments, growth is suspended entirely, such as in cold environments with seasonally frozen soils (Table 1), where the green needles of overwintering confers exhibit very high levels of zeaxanthin [26,27]. Many perennial species adapted to seasonally harsh environments grow incrementally over multiple years—by growing actively during favorable seasons and arresting growth during unfavorable seasons. In an agricultural context, unabated growth is desirable, and floating plants with their fast, continuous growth thus offer attractive opportunities that rival those of terrestrial annuals and biennials.

### 3.3. Plant Source–Sink Balance and the Response to Light, CO_2_, and Nutrient Supply

While both CO_2_ and light are necessary inputs for photosynthesis, the proverbial “too much of a good thing” with respect to CO_2_ and/or light can have negative impacts on plant nutritional quality, growth, and plant lifespan. A report by Myers et al. [57] entitled “Increasing CO_2_ threatens human nutrition” addressed possible adverse effects on plant protein as well as other nutrients. Whereas these impacts on plant nutritional quality for the consumer appear to be rather universal for C_3_ species, other possible impacts of elevated CO_2_ vary among species and with environmental conditions and include possible disruption of photosynthetic productivity as well as accelerated senescence (for a recent review, see [58]). In the following, we examine the possibility that duckweed may be less sensitive to the possible adverse effects of elevated CO_2_ on plant productivity than many other species. We first discuss the effect of elevated CO_2_ on protein (as well some micronutrients) in the context of plant source–sink balance. Photosynthetic organs are the plant’s only source of newly formed carbohydrates, whereas all sugar-consuming tissues are sugar sinks. The maximal capacity of photosynthesis is regulated by the demand for sugar from the plant’s sink tissues [30,59,60]. This regulation by demand is well described for terrestrial plants; excess carbohydrate build up in the leaves leads to downregulation of photosynthetic capacity, which can be the case under conditions of elevated atmospheric CO_2_ and especially in combination with additional environmental factors that cause source–sink imbalances (see, e.g., [61]). *Lemna* exhibits some of the responses described for land plants with respect to the content of protein and micronutrients under very high light and/or CO_2_ supply (see below).

**Figure 6:**
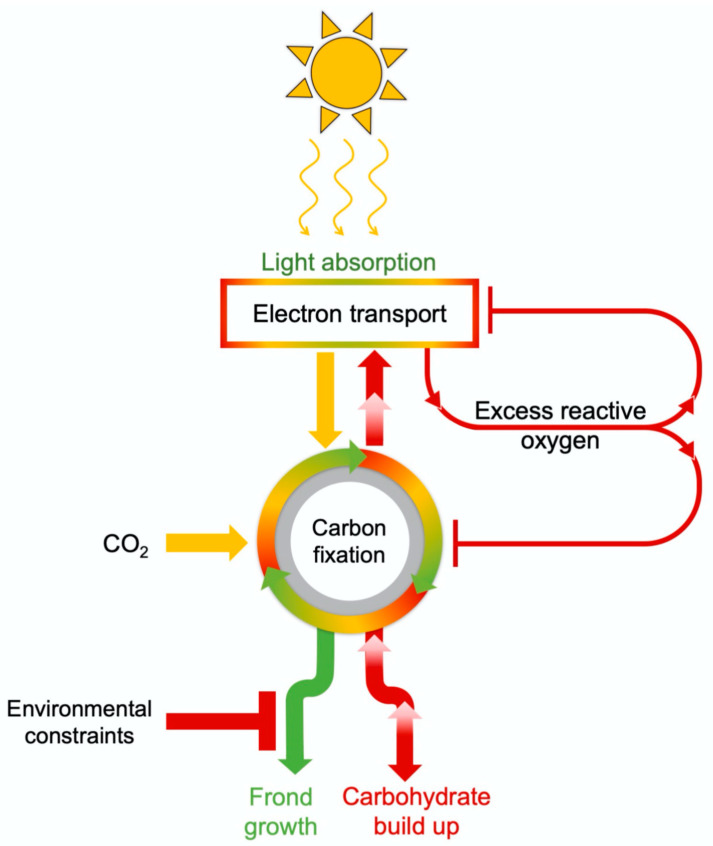
Schematic depiction of the effect of environmental inputs (light, CO_2_, and factors that limit leaf/frond growth) in triggering feedback inhibition of photosynthesis via repression of photosynthetic genes when carbohydrate builds up in leaves/fronds because of limiting sink activity.

Under moderate supply of light and earth-ambient CO_2_, light drives photosynthetic electron transport and allows CO_2_ to be fixed into sugar as the fuel for growth. A combination of high light supply and elevated CO_2_ produces more sugar than is consumed by sink tissues, which leads to feedback inhibition (Figure 6) with build-up of carbohydrate in leaves, backed-up electrons in the photosynthetic electron transport chain, and formation of increased levels of reactive oxygen species (ROS) that are potent repressors of photosynthetic and other genes [62,63,64,65]. An extreme level of foliar carbohydrate build-up and ROS production under elevated CO_2_ can curb the growth of new tissue and accelerate plant senescence [66].

### 3.4. Comparative Evaluation of Plant Response to Light Supply

**Figure 7:**
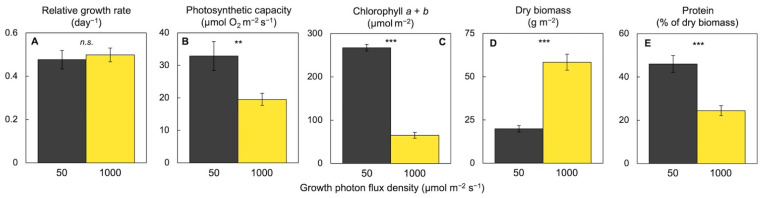
Effect of growth light intensity on (**A**) relative growth rate, (**B**) photosynthetic capacity, (**C**) chlorophyll content, and (**D**) biomass per frond area as well as (**E**) percentage of protein of this biomass for *Lemna gibba*. Data from [23]. *n.s*., not significant. Asterisks indicate significant differences at *p* < 0.01 (**) or *p* < 0.001 (***).

Unlike most terrestrial plants, *Lemna* exhibited the same growth rate in low and high light (Figure 7; [24]), which can be viewed as an absence of growth reductions in low light or as an absence of growth reductions in highly excessive light. In the example of Figure 7, *Lemna* exhibited the same relative growth rate, measured as daily area expansion of new tissue, when grown under low light (50 µmol photons m^−2^ s^−1^) as when grown with a 20× greater light supply (1000 µmol photons m^−2^ s^−1^) for 24 h a day (Figure 7), which exceeds the total daily light supply on the longest, brightest day on earth. However, plants did accumulate more dry biomass (presumably carbohydrate) per frond area under the high light level. Likewise, *Lemna aequinoctialis* fronds growing under continuous light (24 h photoperiod) accumulated more biomass than fronds growing under a 12 h photoperiod of an intermediate light intensity of 200 µmol photons m^−2^ s^−1^ [67].

At the same time, *Lemna* did show some downregulation of maximal photosynthetic capacity (as a correlate of the number of photosynthetic enzymes) as well as pronounced downregulation of light-harvesting capacity (chlorophyll content) under the latter very high light supply (Figure 7; [24]). This downregulation can be viewed as economy on part of a C_3_ plant, whereby greater light exposure and more CO_2_ are able to support the same growth rate (area expansion) and more biomass accumulation with a lesser investment in proteins for light collection and CO_2_ fixation while releasing nitrogen from these photosynthetic proteins for use in area expansion [68]. A consequence of this downregulation is a lower nutritional quality in the form of a lower fraction of the accumulated biomass that consists of protein. The percentage of protein of dry biomass dropped from 46% in low light to under 24% in high light. It has been suggested [69] that the CO_2_-fixing protein ribulose-bisphosphate carboxylase oxygenase may serve in a dual role as not only the CO_2_-fixing enzyme in photosynthesis but also a vegetative storage protein in duckweed fronds. This dual role offers an explanation as to why duckweed vegetative protein would show a response to the demand for carbohydrate from sink tissues.

**Figure 8:**
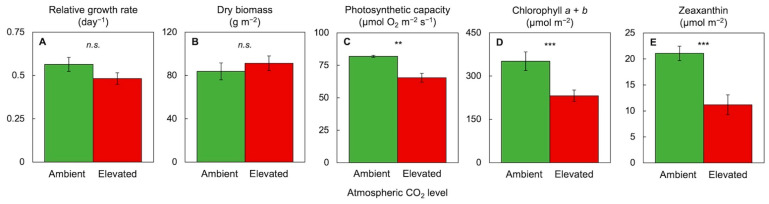
Effect of elevated compared to ambient atmospheric CO_2_ level on (**A**) the relative growth rate (of frond area expansion) as well as (**B**) dry biomass, (**C**) photosynthetic capacity, (**D**) chlorophyll content, and (**E**) zeaxanthin content per frond area in *Lemna minor*. Elevated CO_2_ level was 0.7%; light environment was continuous light of 700 µmol photons m^−2^ s^−1^; nutrient medium was ½ strength Schenk and Hildebrandt medium [72]. For other experimental conditions and methods, see [23]. *n.s*., not significant. Asterisks indicate significant differences at *p* < 0.01 (**) or *p* < 0.001 (***).

**Figure 9:**
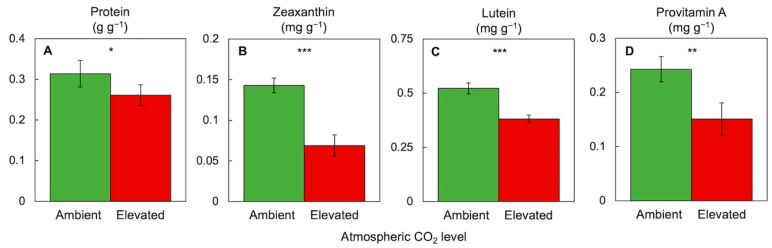
Effect of elevated compared to ambient atmospheric CO_2_ level on various human nutrients including (**A**) protein, (**B**) zeaxanthin, (**C**) lutein, and (**D**) provitamin A (β-carotene) content per dry biomass in *Lemna minor*. CO_2_, light, and nutrient conditions were as listed in the legend of Figure 8. For protein quantification, see [24]. For all other experimental conditions and carotenoid quantification, see [23]. Asterisks indicate significant differences at *p* < 0.05 (*), *p* < 0.01 (**), or *p* < 0.001 (***).

Figure 8 and Figure 9 illustrate responses of *Lemna* duckweed to a combination of high light and elevated CO_2_ with respect to a suite of similar features related to plant productivity, photosynthesis, and nutritional quality for the consumer. Under an extremely high CO_2_ level (0.7%) in the presence of high light (700 µmol photons m^−2^ s^−1^ for 24 h a day) and replete (sufficient) nutrient supply, duckweed grew new tissue at a relative growth rate of frond area expansion with a dry biomass content per area that were both similar under high versus ambient CO_2_ (Figure 8A,B). This finding is consistent with the high growth rate, i.e., high sink activity in the form of new tissue growth, of duckweed, and supports the notion that duckweed is remarkably insensitive to the adverse effects of CO_2_ on photosynthetic productivity. In contrast, there was—as expected—evidence for photosynthetic downregulation in the form of a somewhat lower photosynthetic capacity, much lower chlorophyll content, and much lower zeaxanthin content on a frond area basis (Figure 9) under high versus ambient CO_2_. These findings are consistent with the principle that the plant can support growth and biomass accumulation with less CO_2_-fixing protein in the presence of high CO_2_ levels. Photosynthetic downregulation targets not only the CO_2_-fixing enzymes but also several proteins involved in light collection and processing, including chlorophyll-binding proteins [60]. In turn, lower chlorophyll levels reduce light absorption and the need for the dissipation of excess excitation energy by zeaxanthin and other carotenoids. This downregulation of photosynthesis was thus associated with lower nutritional quality of the plant (Figure 8 and Figure 9) in the form of a lower content of protein, zeaxanthin, lutein, and β-carotene (provitamin A). Lutein [70] and β-carotene [71] have additional roles in plant photoprotection and also act synergistically with zeaxanthin as membrane-soluble antioxidants that fight inflammation in humans [20,21,22].

While *Lemna* is thus susceptible to effects of high CO_2_ that are undesirable for the consumer, it exhibited no decline in the growth of new tissue under conditions of favorable nutrient supply. This relatively modest response to high light and CO_2_ supply may, once again, be associated with the low responsiveness of duckweeds’ growth rate to environmental conditions. Furthermore, we saw no difference in plant response to CO_2_ over a wide range of CO_2_ levels from 0.086% (2 × earth-ambient; not shown) up to 0.7%.

### 3.5. Comparative Evaluation of Plant Response to a Combination of High CO_2_, High Light Supply, and Limiting Mineral Nutrient Supply

Source–sink imbalance is further exacerbated by a combination of elevated CO_2_ and/or high light supply (which increase source strength) with additional environmental factors that decrease sink strength. An example of such an additional factor is a limited mineral nutrient supply [29,73]. The growth of sink tissues is highly sensitive to a shortage of mineral nutrients, especially nitrogen [74]. In terrestrial plants, the combination of elevated CO_2_ and/or high light supply with low nitrogen supply exacerbates source–sink imbalance and downregulates the synthesis of photosynthetic protein [75,76], as well as other metabolic processes [61]. Growth under elevated CO_2_ can also lead to premature leaf senescence when sugars accumulate to levels that inhibit photosynthetic gene expression [77] (see also [75,78,79,80]). Both growth declines and accelerated senescence can be viewed in the context of sugar signals controlling plant progression through the life cycle [81]. Carbohydrate accumulation can be a signal that sufficient resources are available for flowering and completion of the life cycle, and such signals are generated earlier in a plant’s life cycle when limited mineral nutrients enhance carbohydrate accumulation [81]. In fact, it was proposed that some level of nitrogen fertilization of nutrient-limited natural plant communities may mitigate the negative effects of elevated CO_2_ [82].

Duckweeds likewise respond with enhanced carbohydrate accumulation to a combination of high light supply and/or elevated CO_2_ coupled with low nutrient levels in the medium. Pronounced starch accumulation in fronds growing in a nutrient-deficient medium [83,84,85] was further exacerbated by extended photoperiods [86]. However, several traits of duckweeds may lessen the overall impact of such combinations of environmental factors. Duckweeds exhibit a higher tolerance than terrestrial plants to carbohydrate build-up and can accumulate [5] considerable starch levels before showing photosynthetic downregulation [23,83,86,87]. In terrestrial plants, a limited capacity for nitrogen uptake and utilization caused enhanced sink limitation [66,88], whereas a high propensity for efficient nitrogen uptake alleviated sink limitation. Compared to terrestrial plants, *Lemna* exhibits highly efficient uptake [89,90] and conversion of nitrogen to amino acids [91] and accumulates much larger quantities of protein in its fronds [4,83].

Effective nutrient uptake and the propensity to accumulate high protein levels allows duckweed to reclaim polluted waters [6,8] and convert wastewater to high-quality animal feed [5,7,9,92]. The quantity of edible protein produced by duckweed per hectare of production area greatly exceeds that of soybean [5,93] since duckweeds accumulate high levels of vegetative storage protein in their fronds [4,93], whereas soybean accumulates high levels of protein only in its seeds, which represent a small fraction of the plant. Highly efficient nutrient uptake and accumulation of vegetative storage protein, resulting in exceptionally high plant protein contents, is also seen in other aquatic floating plants [94,95]. Many of these other aquatic plants are also edible; more than 70 wetland plants of India were identified as edible or medicinal plants by Swapna et al. ([38], e.g., ten species in the Asteraceae, six each in the Poaceae and Commelinaceae, and six in the order Alismatales, with four Araceae and two Hydrocharitaceae; see also [96]). From an ecological perspective, a combination of effective uptake and the storage of nitrogen as vegetative storage protein in aquatic environments may be advantageous in small- to mid-sized freshwater bodies that receive an intermittent influx of nutrients. Duckweeds were shown to continue growing in low-nutrient media for a certain amount of time by utilizing internal nutrient stocks [83]. Efficient nutrient uptake and storage, as vegetative protein, should enable the plants to take advantage of the pulses of nutrient availability in support of the continuous growth of new fronds until dense duckweed mats dramatically reduce the light available to algae and submerged aquatic plants that compete for nutrients. The properties shared by aquatic floating plants that set them apart from most terrestrial plants thus reflect adaptations to the unique aquatic environment with its inherent variability and opportunities.

### 3.6. Comparative Evaluation of Plant Response to Combinations of High CO_2_, High Light Supply, and High Mineral Nutrient Supply

Remarkably, high nitrate supply can also decrease photosynthetic rates of terrestrial plants, especially when combined with conditions that result in carbohydrate build-up, such as extended photoperiods [97] or elevated CO_2_ [98,99]. Under these conditions, excessive ROS production in the chloroplast is combined with the production of additional oxidants in other cell compartments that participate in nitrate metabolism. Specifically, a high activity of the nitrate-reducing enzyme nitrate reductase produces high levels of messengers (both ROS and reactive nitrogen species, RNS; [99]) that modulate nitrogen metabolism [100,101,102] and can also trigger plant senescence ([66,103]; see also [104,105]). Such findings led to a suggestion that “in a future environment with high CO_2_ levels the use of fertilizers containing low concentrations of nitrate could improve … [nitrogen] assimilation” in terrestrial crops [106]. A similar warning and recommendations were issued by Bloom [107] in a communication entitled “As carbon dioxide rises, food quality will decline without careful nitrogen management.” Ammonium metabolism does not have the same propensity as nitrate metabolism for the generation of ROS and RNS [108] and the resulting disruption of redox homeostasis. On the other hand, the accumulation of ammonium in plant tissue can disrupt cellular pH balance (and some other aspects of metabolism) in many terrestrial plants [109]. However, some plants, including those growing in marshes, are adept at using ammonium [107]. The preference of duckweeds (and other aquatic plants) for the uptake of ammonium over nitrate also has the potential to avoid high rates of nitrate reduction and its adverse effects, and duckweed’s efficient conversion of ammonium to vegetative storage protein limits ammonium accumulation and resulting toxicity.

## 4. Plant–Microbe Interaction and the Abiotic Environment

Whereas adverse effects of high CO_2_ in the absence of nutrient deficiency or other abiotic stresses can presumably be mitigated by avoiding high light intensities or long photoperiods, the additional presence of unfavorable nutrient conditions will likely require other measures. Plant–microbe interaction may offer the mitigation of adverse effects of combinations of environmental factors that exacerbate the source–sink imbalance. The extent of plant response to the presence of microorganisms depends on environmental conditions [110] such as CO_2_ level (see, e.g., [111,112,113,114]) and nitrogen availability (see, e.g., [115,116]). *Lemna minor* exhibited a decline in growth rate, which was strongly exacerbated under elevated CO_2_ levels (twice-ambient), starting several days following the transfer from replete nutrient medium to very low nutrient levels in Schenk and Hildebrandt [72] medium diluted by a factor of 1/20 [117].

While experimental manipulation of the plant microbiome is challenging in terrestrial plants growing in soil, some evidence is available for enhanced plant productivity in the presence of fungal partners of terrestrial plants [118,119]. Inoculation of terrestrial plant roots with arbuscular mycorrhizal fungi increased root volume and activity, triggering photosynthetic upregulation in some systems [118,120]. Conversely, the elimination of the mycorrhizal system of cucumber resulted in declining photosynthesis rates ([121]; see also [122]). Transcriptomic analysis of such systems revealed differential gene expression in pathways of photosynthesis, hormone metabolism, carbohydrate metabolism, amino acid metabolism, stress response, signal transduction, and antioxidation [119].

Legumes that feature symbioses with N_2_-fixing bacteria had greater photosynthesis rates under elevated CO_2_ and maintained higher protein content and higher overall ratios of nitrogen to carbon in their tissues [123]. In contrast, most non-leguminous C_3_ crops exhibit increased carbon-to-nitrogen ratios under elevated atmospheric CO_2_ [124,125]. Lemnaceae exhibit robust interaction with microorganisms [126,127,128], and duckweed photosynthesis rate as well as growth rate can be stimulated by the plant microbiome [127,128,129,130,131,132]. Whereas plant–microbe interaction can thus clearly benefit the plant, the existence of additional layers of complexity should be noted. Microorganisms may engage in competition with plants for mineral nutrients in certain environments; the same bacterial strains that strongly promoted duckweed growth under some conditions reduced plant growth under limiting levels of mineral nutrients other than nitrogen [127,130,133]. Duckweeds are an attractive model system for the study of plant–microbe interaction due to the ease of inoculation and manipulation of the rhizosphere as well as the fact that their small size and high growth rates favor multi-factorial design of growth experiments in different growth environments with plants fully acclimated to these conditions. Other aquatic floating plants also form alliances with microorganisms, especially potential N_2_-fixing clades (see, e.g., [134]).

**Figure 10:**
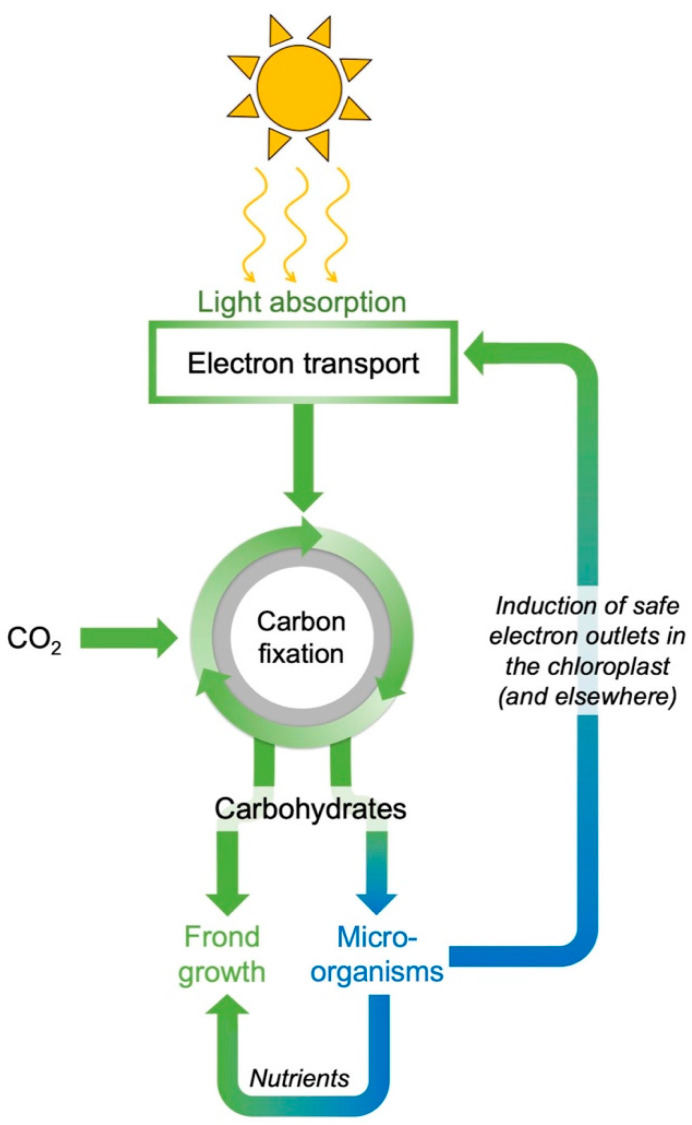
Schematic depiction of the effect of microorganisms in counteracting feedback inhibition of photosynthesis by (i) acting as a sink for carbohydrates, (ii) improving plant nitrogen content in support of new growth (as well as photosynthetic capacity), and (iii) indirectly and directly promoting safe electron outlets in chloroplasts and elsewhere.

**Figure 11:**
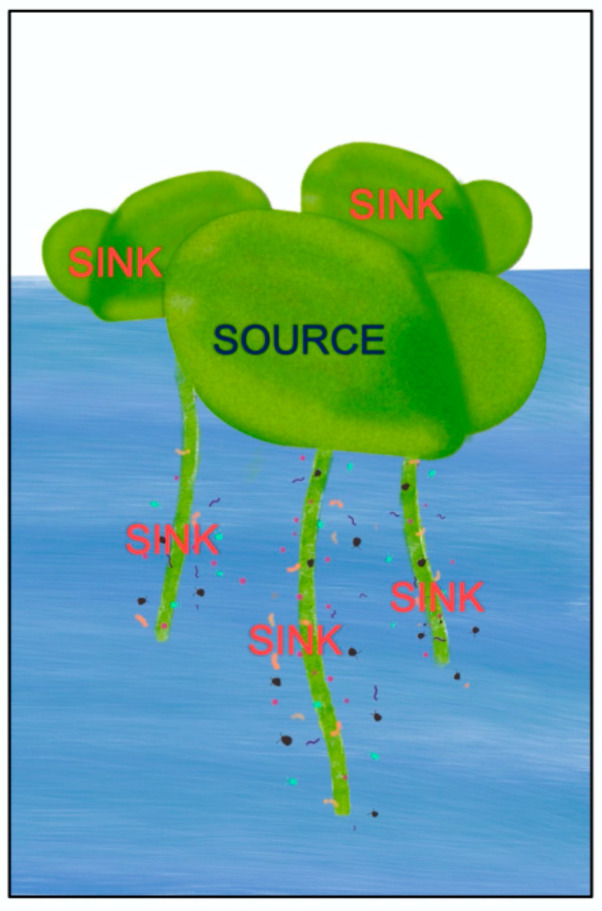
Schematic depiction of a *Lemna* colony consisting of a mother frond as the source of photosynthate for expanding daughter fronds as well as for the rhizosphere microbiome as sinks of photosynthate.

An integrative review of literature on (i) plant performance under high CO_2_ and (ii) plant–microbe interaction and symbioses between photosynthetic and non-photosynthetic organisms [58] suggests that microorganisms can enhance plant photosynthetic productivity and nutritional quality (Figure 10 and Figure 11) by (i) serving as an additional sugar sink that prevents carbohydrate build-up [130,132,135,136,137,138] and resulting excess ROS formation (see above), (ii) balancing nutrient supply (limiting or excess; see above) and producing growth factors [139], (iii) producing gene regulators that safely re-route electrons and, thereby, further counteract disruption of redox homeostasis under both limiting nutrient supply and very high nitrate supply (see, e.g., [108,140]). Alternative outlets for electrons include cyclic electron flow in the chloroplast [141,142,143,144,145,146]. Since mitochondria and cell membrane-associated processes also produce excess oxidants under elevated CO_2_ levels, the maintenance of cellular redox homeostasis [64] requires coordination of electron flow in chloroplasts and mitochondria. Plant-specific mitochondrial alternative oxidase (AOX) is a key player in this coordination [147,148,149,150,151] and serves as a safe outlet for electrons when environmental shifts threaten to disrupt metabolism [147,152,153]. Plant AOX levels increased in response to high CO_2_ and light supply [147,148,154] as well as under limiting nitrogen [155]. Furthermore, the plant rhizosphere microbiome interacts with plant AOX [140].

Plant–microbe interaction may thereby allow plants to take advantage of high light and CO_2_ for growth and biomass accumulation by maintaining a high photosynthetic capacity with high levels of photosynthetic protein, chlorophyll, and chlorophyll-protecting antioxidants. This suggestion is supported by recent findings that the inoculation of previously sanitized *L. minor* with pond water supporting populations of *L. minor* prevented the decline in relative growth rate seen upon transfer to 1/20 strength Schenk and Hildebrandt medium in un-inoculated controls [117]. Moreover, inoculated fronds accumulated greater levels of dry biomass with the same ratio of protein to biomass as uninoculated fronds under either ambient or twice-ambient CO_2_ [117]. By supporting a sustained high photosynthetic capacity, microorganisms can thus favor the production of a steady stream of carbohydrate for consumption by both microorganisms and growing plant tissue and discourage metabolic imbalances that trigger photosynthetic downregulation and/or accelerated senescence.

## 5. Conclusions

The unique features of aquatic plants reflect adaptations to the freshwater environment and include rapid growth, high productivity, and strong accumulation of high-quality vegetative storage protein as well as essential human micronutrients. These micronutrients include carotenoids with wide-ranging roles in human health and a unique carotenoid composition of *Lemna*. Other aquatic plants may share this latter trait and should be further tested for edibility and nutritional quality. The relative insensitivity of duckweed’s growth rate to environmental conditions and plant source–sink imbalance may allow duckweeds to take advantage of elevated atmospheric CO_2_ levels via particularly strong stimulation of biomass production and relative insensitivity to declines in the growth of new tissue. It may be possible to mitigate the declines in nutritional quality under elevated CO_2_ associated with regulatory adjustments in photosynthetic metabolism by plant–microbe interaction.

## Figures and Tables

**Table 1:** Water-acquisition strategies, growth patterns, and species examples that illustrate the continuum of plant adaptations to water availability.

Habitat	Water-Acquisition Strategy	Growth Pattern	Example
Aquatic plants	Continuous access to water body until it dries up	Fast growth	Lemnaceae
Terrestrial plants	Continuous access to water until source dries up	Very fast growth and life cycle completion	Desert ephemerals
	Continuous access to water, increased root volume, osmotic adjustment	Relatively fast growth throughout life cycle	Annuals and biennials
	Continuous access to water table	Steady growth throughout the seasons	Palm and mesquite
	Enhanced acquisition of minimal soil water via large root volume, osmotic adjustments	Slow growth despite minimal soil water	Desert shrub *Encelia*, creosote bush
	Storage of water in plants	Very slow growth	Succulents, cacti
	Tolerance of seasonal loss of water	Seasonal complete growth arrest	Conifers in frozen soil

## Data Availability

Data are contained within the article.
